# Infectious optic neuropathy: the interplay between pathogens and the host immune system—a review of diagnostic and therapeutic dilemmas

**DOI:** 10.3389/fcimb.2026.1896851

**Published:** 2026-08-13

**Authors:** Yin Jiang, Meng Si, Fan Chen, Bei Chen, Na Long, Fei Jiang, Shangang Pan, Xueqin Chu, Yanming Tian, Huarong Wu

**Affiliations:** 1 Anqing Municipal Hospital, Anqing, China; 2 Anqing Shihanbin Aier Eye Hospital, Anqing, China; 3 Southwest Medical University Affiliated Tianfu Hospital, Meishan, China

**Keywords:** clinical decision-making, corticosteroids, infectious optic neuropathy, mNGS, MOGAD, post-infectious immune-mediated injury

## Abstract

Infectious optic neuropathy (ION) represents a major clinical challenge at the intersection of ophthalmology and neurology. Its pathogenesis typically involves a complex interplay between direct pathogen invasion and post-infectious immune-mediated injury. Current clinical management faces two core challenges: (1) accurately differentiating direct pathogen-induced injury from post-infectious autoimmune optic neuritis [e.g., myelin oligodendrocyte glycoprotein antibody-associated disease (MOGAD)]; and (2) avoiding exacerbation or dissemination of occult infection when immunosuppressive therapy is required. Traditional static classification models based on pathogen profiles are insufficient to guide dynamic clinical decision-making and may lead to delayed treatment or overtreatment. In this review, we propose a dynamic decision-making framework grounded in pathophysiological mechanisms. We summarize practical clinical clues for distinguishing these two injury patterns, outline key considerations for systemic corticosteroid use, and discuss the positioning of emerging diagnostic technologies—such as metagenomic next-generation sequencing (mNGS)—within current clinical pathways. Drawing on available clinical evidence, we advocate an individualized intervention strategy centered on “dynamic balance,” emphasizing that decisions should be guided by the predominant mechanism at each disease stage rather than a rigid dichotomy between “infectious versus non-infectious.” Finally, we highlight key evidence gaps and underscore that this mechanism-informed framework is a pragmatic synthesis that requires prospective validation.

## 1 Introduction

Although infectious optic neuropathy (ION) is relatively uncommon in clinical practice, it carries a high risk of severe visual loss and blindness. The burden is particularly pronounced in immunocompromised hosts, including individuals with human immunodeficiency virus (HIV) infection, organ transplant recipients, and patients receiving long-term immunosuppressive therapy. A compromised immune defense renders the optic nerve susceptible to pathogen invasion, potentially resulting in irreversible visual impairment. Common causative pathogens include cytomegalovirus (CMV), varicella-zoster virus (VZV), Treponema pallidum, Mycobacterium tuberculosis, and various fungi. Other clinically relevant causes include Bartonella henselae and Borrelia burgdorferi, which may present with optic nerve involvement and further broaden the differential diagnosis (Ambika and Lakshmi, 2024). Early manifestations often overlap substantially with those of idiopathic or demyelinating optic neuritis, making differential diagnosis challenging. In people living with HIV, the cluster of differentiation 4 (CD4)^+^ T-cell count and viral load are key biomarkers for distinguishing infectious from non-infectious optic nerve involvement (Jindahra et al., 2020).

Atypical phenotypes associated with specific pathogens further complicate diagnostic confirmation. For example, posterior uveitis due to T. pallidum may be mistaken for acute retinal necrosis and often requires multimodal imaging to differentiate it (Pichi and Neri, 2020). Importantly, syphilitic optic neuropathy may also present in isolation without concomitant uveitis, which can increase the risk of misclassification in early evaluation (Schulz et al., 2021). Ocular tuberculosis (accounting for approximately 1% of patients with tuberculosis) can also involve the optic nerve, and early standardized anti-tuberculosis therapy can significantly improve visual prognosis (Jiang et al., 2021). In addition, fungi such as Cryptococcus neoformans can induce optic neuritis even in immunocompetent hosts (Kathiresu et al., 2020). Paradoxical inflammatory worsening may occur during treatment in selected settings; in immunocompromised individuals undergoing immune recovery, this phenomenon is referred to as immune reconstitution inflammatory syndrome (IRIS) and may lead to secondary clinical deterioration (Kathiresu et al., 2020). Because misdiagnosis or delayed intervention can lead to irreversible vision loss, establishing a precise differential diagnostic workflow is essential.

At present, clinical management faces dual challenges in both diagnosis and intervention. From a diagnostic perspective, direct pathogen-mediated injury and post-infectious immune-mediated injury may evolve sequentially or coexist. Moreover, conventional imaging findings—such as optic nerve enhancement patterns on magnetic resonance imaging (MRI)—and optical coherence tomography (OCT) parameters (e.g., ganglion cell layer thickness) often overlap across these mechanisms, making a single diagnostic modality insufficient for etiologic attribution. Clinical presentations are also highly heterogeneous across pathogens. Chikungunya virus infection or yellow fever vaccination can induce acute disseminated encephalomyelitis (ADEM), with extensive white matter lesions involving the optic nerve and central nervous system (Carvalho et al., 2020). Leptospirosis may present with isolated optic neuritis without specific features (Rajput et al., 2021). Conversely, neurotoxocariasis can manifest as meningoencephalitis with optic nerve involvement, and confirmation typically requires the integration of cerebrospinal fluid eosinophilia and serologic evidence (Meliou et al., 2020). Post-infectious autoimmune optic neuritis, such as myelin oligodendrocyte glycoprotein antibody-associated disease (MOGAD), represents an additional diagnostic pitfall because it can mimic infection-related inflammation in the acute setting (Rempe et al., 2021). Notably, pediatric and adult patients may differ in immune maturation and exposure patterns, which can influence the likelihood of post-infectious immune phenotypes (e.g., MOGAD) and the pretest probability of specific etiologies; therefore, diagnostic clues and treatment decisions should be interpreted in an age- and context-aware manner (Pihl-Jensen et al., 2021; Schulz et al., 2021; Li et al., 2023).

Therapeutically, systemic corticosteroids are the standard of care for demyelinating optic neuritis; however, their use during active infection can suppress host defenses and promote pathogen dissemination (Alcibahy et al., 2024). This risk is particularly salient in CMV retinitis, where inappropriate corticosteroid exposure may accelerate retinal necrosis. In contrast, data on tuberculous optic neuropathy suggest that, when adequate anti-tuberculosis therapy is in place, adjunct oral corticosteroids may improve overall outcomes (Jiang et al., 2021). In the absence of a pathophysiology-based classification standard, clinicians are often forced to decide whether to prioritize antimicrobial therapy or anti-inflammatory treatment.

Accordingly, rather than attempting to exhaustively catalogue all pathogens and rare case reports, this review proposes a dynamic clinical decision-making framework based on pathophysiological mechanisms. Our central premise is that treatment strategies should not be constrained by an absolute dichotomy between “infectious” and “non-infectious,” but should instead be guided by the predominant injury pattern at a given stage of disease. Clinical observations suggest that such switching can occur across the disease course. For instance, in people living with HIV, non-infectious optic neuritis emerging during CD4^+^ recovery and viral suppression is largely driven by IRIS-mediated immune injury (Jindahra et al., 2020). Severe acute respiratory syndrome coronavirus 2 (SARS-CoV-2) infection has been reported to trigger Vogt–Koyanagi–Harada (VKH)-like bilateral granulomatous panuveitis with optic neuritis, which may require corticosteroids and immunomodulatory agents for disease control (Santamaria et al., 2022). Similarly, typical bacterial infections, such as Klebsiella pneumoniae, can induce para-infectious optic neuritis that responds to high-dose methylprednisolone pulse therapy in reported cases (Wang Q. et al., 2024). Building on these observations, we summarize clinical clues for recognizing the two core injury patterns, delineate four major clinical scenarios for corticosteroid decision-making, and discuss the role of emerging diagnostic technologies such as metagenomic next-generation sequencing (mNGS) within current clinical pathways, aiming to provide a scientifically grounded and clinically feasible decision framework.

## 2 Pathophysiological mechanisms

The onset and evolution of ION are not driven by a single pathway; rather, they represent a dynamic process in which direct pathogen-mediated injury intertwines with dysregulated host immune responses. Depending on pathogen characteristics, baseline immune status, and disease stage, two major mechanistic patterns predominate: (1) direct tissue injury caused by active microbial replication and (2) autoimmune attack triggered after pathogen containment through mechanisms such as molecular mimicry. Accurate differentiation between these mechanisms—and recognition of their context-dependent transitions—is essential to avoid the therapeutic paradox of “antimicrobial versus anti-inflammatory” treatment in clinical practice.

### 2.1 Tissue-destructive pattern driven by direct pathogen invasion

Direct pathogen invasion is the classic pathogenic pathway and is most often observed in immunocompromised hosts, such as individuals with HIV infection, organ transplant recipients, and patients receiving long-term immunosuppressive therapy. The defining feature is active replication of microorganisms within the optic nerve and adjacent tissues, leading to cytolysis, necrotizing inflammation, or microabscess formation. For example, necrotizing retinitis with optic nerve involvement caused by herpesviruses such as CMV constitutes an ophthalmic emergency (Devilliers et al., 2021). Filamentous fungi (e.g., Aspergillus and Mucor) may directly penetrate the optic nerve parenchyma and cause profoundly destructive pathology (Ambika and Lakshmi, 2024). T. pallidum can also proliferate locally, forming gummatous inflammatory granulomas that involve the optic nerve (Schulz et al., 2021). Notably, direct invasion by certain pathogens may phenotypically mimic autoimmune disease. In murine models, Zika virus has been reported to replicate persistently in ocular tissues, and the resulting clinical features may resemble neuromyelitis optica spectrum disorder (NMOSD)-like manifestations; however, these findings primarily reflect direct viral infection and tissue injury in preclinical settings rather than confirmed human autoimmune pathophysiology (Brainer-Lima et al., 2021).

The key clinical implication of this mechanism is that effective intervention relies primarily on adequate, pathogen-sensitive antimicrobial therapy. In individuals with HIV co-infected with cryptococcal meningitis, an extremely low CD4^+^ T-cell count (median, 36.5 cells/µL) together with a high viral load often indicates uncontrolled pathogen activity, warranting prompt targeted therapy with amphotericin B plus fluconazole (Jindahra et al., 2020). Similarly, in patients with poorly controlled diabetes, destructive Klebsiella-associated panophthalmitis with optic nerve involvement may occur (Kashkash et al., 2023). At this stage, the premature introduction of immunosuppressive agents (e.g., systemic corticosteroids) can increase the risk of pathogen dissemination and is generally contraindicated unless used with great caution and accompanied by robust, effective antimicrobial coverage.

### 2.2 Secondary inflammatory pattern mediated by post-infectious immunity

**Figure 1:**
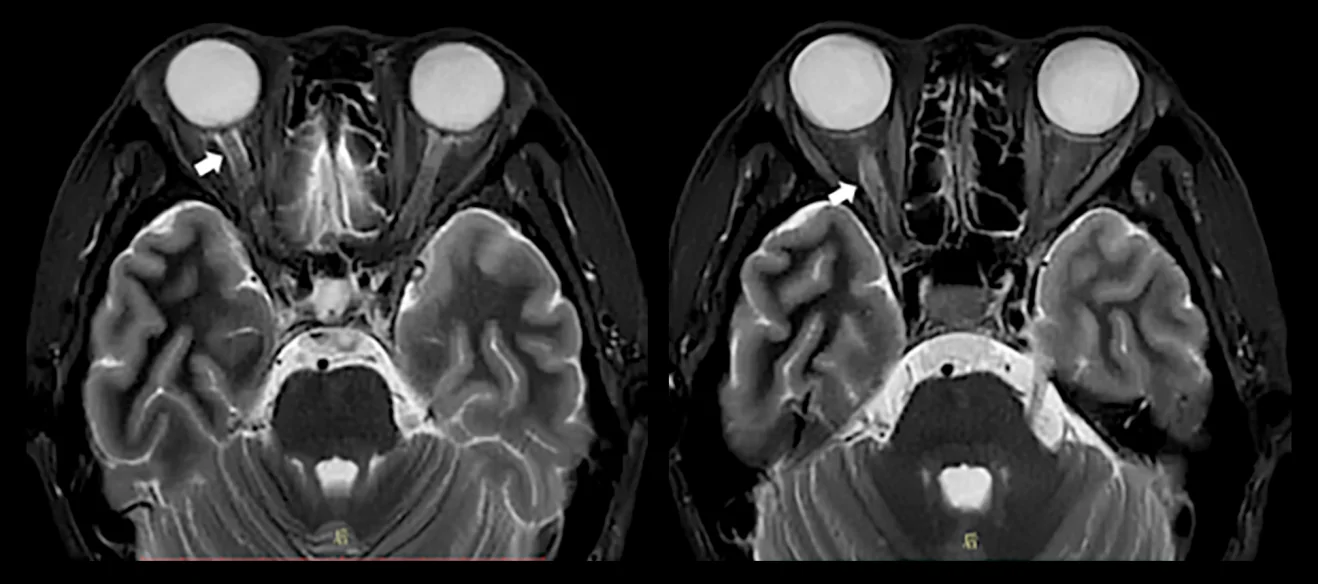
Neuroimaging hallmarks of post-infectious immune-mediated optic neuritis (MOGAD). Axial T2-weighted orbital magnetic resonance imaging (MRI) images from a 42-year-old woman with vision loss and seropositivity for MOG-IgG. The images (two views from the same sequence) demonstrate high-signal intensity involving the optic nerve sheath around the right optic nerve (white arrows), forming a typical “double track sign,” consistent with optic perineuritis reported in MOGAD-associated optic neuritis. This real-world example supports the role of neuroimaging in recognizing immune-mediated phenotypes in the appropriate clinical and serologic context. [Reprinted from (Wang MM. et al., 2024), under CC BY-NC-ND 4.0]. IgG, immunoglobulin G; MOG-IgG, myelin oligodendrocyte glycoprotein IgG; MOGAD, myelin oligodendrocyte glycoprotein antibody-associated disease; MRI, magnetic resonance imaging.

The post-infectious immune-mediated injury pattern is more common in immunocompetent hosts and has attracted increasing attention following the coronavirus disease 2019 (COVID-19) pandemic. Multiple studies suggest that autoimmunity and molecular mimicry may contribute to COVID-19–related neuro-ophthalmic sequelae (Priyal et al., 2023). In this pattern, although the invading pathogen has been cleared or suppressed to a very low burden, the host immune system continues to attack self-tissues through effects such as molecular mimicry or bystander activation. Molecular mimicry refers to substantial structural similarity between pathogen epitopes and central nervous system autoantigens, such as myelin oligodendrocyte glycoprotein (MOG) or aquaporin-4 (AQP4). Sequence homology analyses have suggested that the Zika virus NS5 protein is similar to the host proteolipid protein (PLP), raising the hypothesis of molecular mimicry–related demyelinating disease; however, this remains a proposed mechanism rather than an established human pathophysiology (França et al., 2023). Similarly, case-based evidence has described Epstein–Barr virus (EBV) reactivation as a potential cofactor in MOG antibody–associated optic neuritis, but causality and generalizability require further study (Georgopoulou et al., 2024). Anatomically, such post-infectious immune responses often manifest with distinct neuroimaging hallmarks, such as profound perineuritis, which reflect the underlying inflammatory cascade targeting the optic nerve sheath (**Figure 1**).

In addition, rare case reports have described AQP4 antibody–positive NMOSD following COVID-19 vaccination in susceptible individuals, which should be interpreted as an association rather than as definitive evidence of a causal mechanism (Uchida et al., 2026). Under this mechanistic pattern, the therapeutic focus fundamentally shifts: immunomodulation, rather than antimicrobial therapy, becomes the core strategy. Case reports and small series suggest that, whether in bilateral optic neuritis following influenza A(H1N1) infection (Ambika et al., 2020) or in ADEM secondary to COVID-19 with concomitant optic nerve injury (Rossi et al., 2022), visual outcomes may improve with early initiation of high-dose methylprednisolone pulse therapy and intravenous immunoglobulin (IVIG). Therefore, when optic neuropathy emerges in the later phase after infection in immunocompetent individuals, immune-mediated injury should be considered, and an immunomodulatory treatment pathway may be appropriate when active infection has been reasonably excluded. As a clinical note, pediatric and adult patients may differ in the pretest probability of post-infectious immune etiologies (e.g., MOGAD), which should be considered during diagnostic stratification; treatment risk–benefit considerations may also warrant age- and context-aware individualization (Wendel et al., 2020; Li et al., 2023; Wang MM. et al., 2024).

### 2.3 Spatiotemporal overlap and mechanistic transition

In clinical practice, these two mechanisms are neither isolated nor static. A single patient may sequentially experience direct pathogen invasion and post-infectious immune-mediated injury, and such transitions can manifest as paradoxical inflammatory worsening. In immunocompromised hosts undergoing immune recovery—most classically in people living with HIV after initiation of antiretroviral therapy (ART)—this phenomenon is termed IRIS. In contrast, paradoxical reactions can also occur in immunocompetent patients (e.g., in tuberculosis), and IRIS should not be used interchangeably with paradoxical reaction in these settings (Quinn et al., 2020). Moreover, in infections such as tuberculosis, overlapping contributions from pathogen-driven injury and host inflammatory responses may be present even before treatment initiation, highlighting that “overlap” is not exclusively a treatment-triggered phenomenon (Comez, 2019). During paradoxical worsening or IRIS, despite a decline in pathogen burden, changes in host immune regulation—particularly a rapid rebound of antigen-specific lymphocyte responses—can trigger an intense inflammatory response against residual microbial antigens. Among individuals with HIV, although ART increases CD4^+^ T-cell counts and suppresses viral replication, some patients develop non-infectious optic neuritis in which IRIS-driven immune injury predominates (Jindahra et al., 2020). Similarly, paradoxical inflammatory reactions with optic nerve involvement have been reported during treatment in immunocompetent patients with cryptococcal meningitis; these presentations are sometimes described as “IRIS-like,” but they should be distinguished from classic IRIS, which occurs after immune restoration in immunosuppressed hosts (Kathiresu et al., 2020).

The essence of this transformation is that, while antimicrobial therapy mitigates direct pathogen-mediated injury, shifts in the host immune milieu—particularly in specific contexts such as HIV-associated immune dysregulation and subsequent immune recovery—may facilitate excessive inflammatory activation, including autoreactive T- and B-cell responses. Clinicians should therefore move beyond static, label-based diagnoses and adopt real-time monitoring of disease evolution. In people living with HIV, rising peripheral CD4^+^ T-cell counts together with declining pathogen nucleic acid load represent key markers for distinguishing infectious from non-infectious optic nerve injury (Coutel et al., 2022). In addition, cerebrospinal fluid cytology, quantitative protein assessment, and oligoclonal band (OCB) analysis provide important ancillary support for differential diagnosis (Vlad et al., 2023). In the setting of dynamic mechanism switching, clinical decision-making should be proactive: while maintaining adequate antimicrobial coverage, immunomodulatory agents may need to be introduced in selected cases to attenuate excessive inflammatory cascades.

## 3 Diagnostic strategies

Traditional evaluation of suspected ION often follows a passive “diagnosis of exclusion” approach: infectious etiologies are considered only after demyelinating diseases such as multiple sclerosis (MS) and NMOSD have been ruled out through clinical assessment and imaging. However, this delayed strategy is inefficient and carries substantial risk. Because infectious phenotypes frequently overlap with autoimmune inflammation, relying solely on exclusion can readily lead to misdiagnosis. For example, Toxoplasma infection may present as papillitis or neuroretinitis, typically with subacute unilateral visual loss and pain on eye movements; symptoms, MRI findings, and even cerebrospinal fluid profiles may mimic demyelinating disease (Pihl-Jensen et al., 2021). If the etiology is misclassified and immunosuppressive therapy is initiated indiscriminately, fulminant dissemination of the pathogen may ensue.

**Figure 2:**
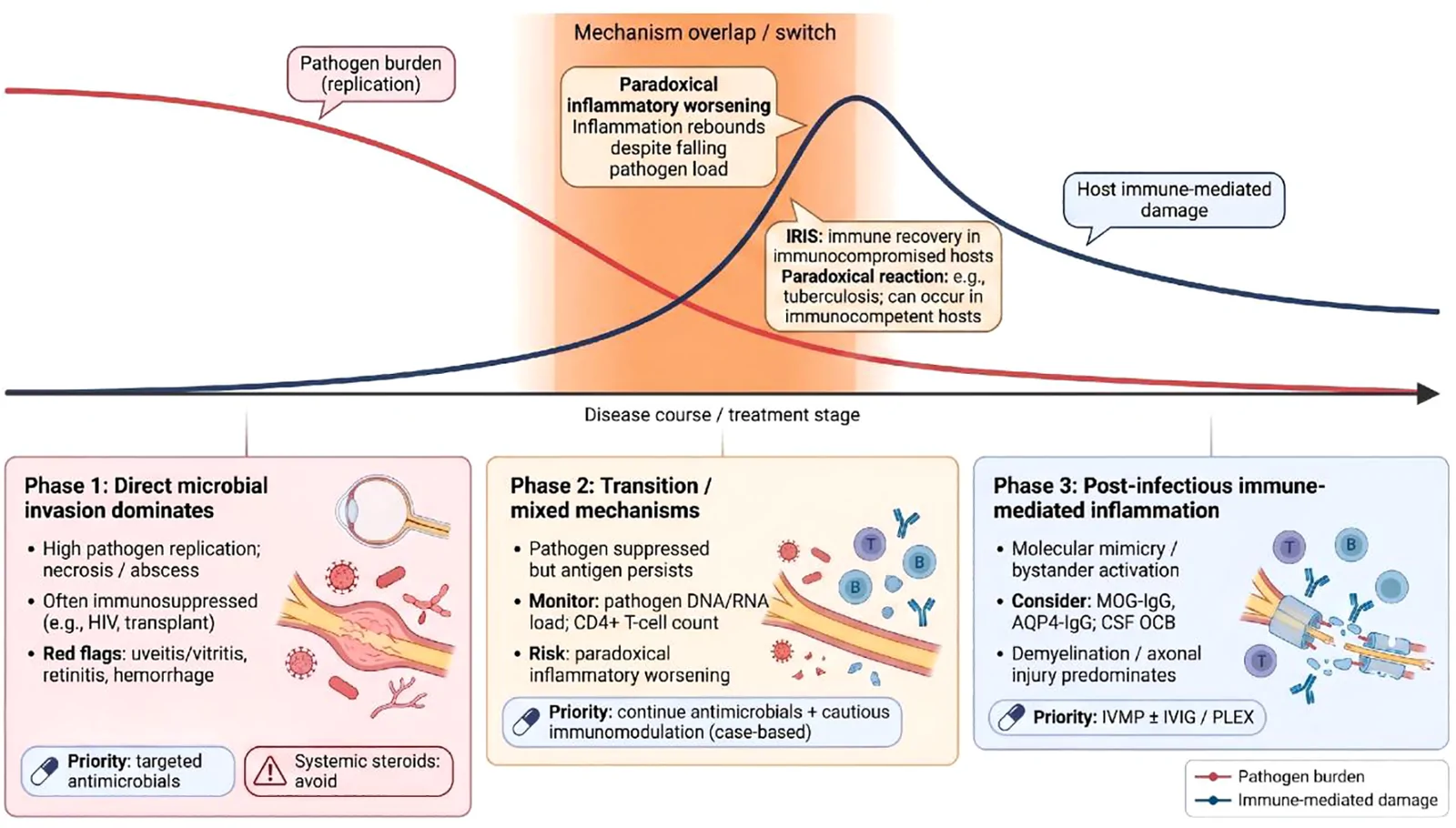
The mechanistic continuum of infectious optic neuropathy (ION). The schematic depicts a stage-dependent continuum in which the predominant driver of optic nerve injury shifts between pathogen burden/replication and host immune-mediated damage. Phase 1 is dominated by direct microbial invasion with high pathogen replication and tissue-destructive inflammation, prioritizing prompt targeted antimicrobials and generally avoiding systemic corticosteroids. Phase 2 represents a transition/mixed-mechanism stage in which pathogen replication may be suppressed yet antigenic stimulation persists, creating risk for paradoxical inflammatory worsening (inflammation rebounds despite falling pathogen load). This includes immune reconstitution inflammatory syndrome (IRIS) in immunocompromised hosts undergoing immune recovery, whereas paradoxical reactions can also occur in immunocompetent settings (e.g., tuberculosis). Phase 3 reflects post-infectious immune-mediated inflammation (e.g., molecular mimicry/bystander activation and demyelination/axonal injury), in which immunotherapy is typically considered after reasonable exclusion of active infection. (Schematic generated with the assistance of Gemini 3.1 image editor). ION, infectious optic neuropathy; IRIS, immune reconstitution inflammatory syndrome; HIV, human immunodeficiency virus; DNA, deoxyribonucleic acid; RNA, ribonucleic acid; CD4, cluster of differentiation 4; CSF, cerebrospinal fluid; OCB, oligoclonal bands; MOG-IgG, myelin oligodendrocyte glycoprotein IgG; AQP4-IgG, aquaporin-4 IgG; IVMP, intravenous methylprednisolone; IVIG, intravenous immunoglobulin; PLEX, plasma exchange.

Accordingly, contemporary diagnostic strategies should move beyond passive exclusion and instead proactively seek dual evidence for infection and autoimmunity. An upfront, tiered assessment pathway can help avoid delays in antimicrobial therapy while minimizing the risk of inappropriate immunosuppression. To summarize the dynamic predominance, overlap, and transitions—including paradoxical inflammatory worsening (e.g., IRIS in immunocompromised hosts)—between pathogen burden and immune-mediated injury across the disease course, we provide a mechanistic continuum schematic (**Figure 2**). **Figure 2** is intended as a conceptual aid to guide time-sensitive decisions by identifying which mechanism is likely predominant at a given stage, rather than as a rigid diagnostic label.

### 3.1 Infection-driven pathophysiologic clues

**Figure 3:**
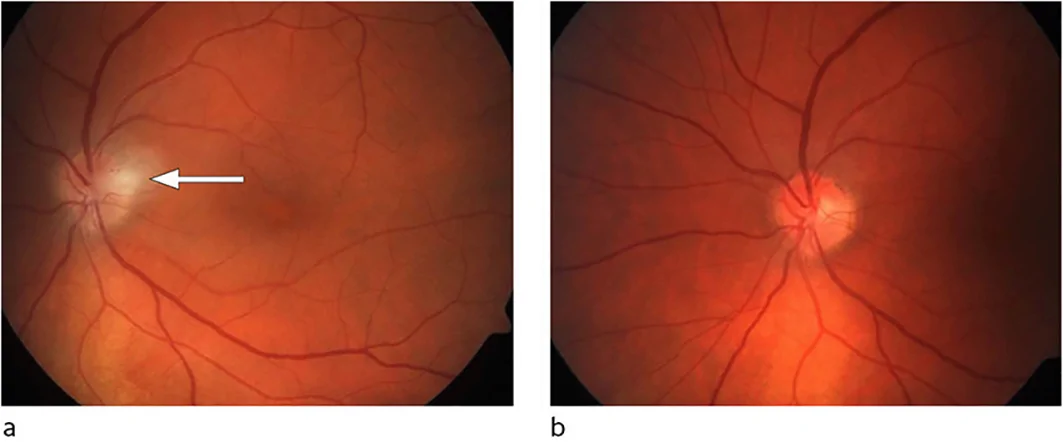
Clinical mimicry and infectious “red flag” signs in ocular toxoplasmosis. **(A)** Fundus photograph of the left eye at presentation, showing marked unilateral optic disc swelling that can mimic acute demyelinating optic neuritis. The presence of a subtle whitish peripapillary infiltrate (white arrow) represents an infection-driven “red flag” that should prompt consideration of alternative etiologies and careful corticosteroid decision-making. **(B)** Follow-up fundus photograph after six weeks of pathogen-directed antimicrobial therapy, showing interval improvement of optic disc edema and the peripapillary infiltrate. [Reprinted from (Pihl-Jensen et al., 2021), under CC BY-ND 4.0].

“Red flag signs” are critical clinical markers for early recognition of direct pathogen invasion, encompassing ocular findings, systemic manifestations, and imaging features. Ocular red flags include concomitant uveitis, marked vitreous opacity, progressive retinal necrosis (e.g., the classic “pizza pie” lesions of CMV retinitis), diffuse peripapillary hemorrhages, and macular edema, which are suggestive of an infectious etiology (Devilliers et al., 2021; Standardization of Uveitis Nomenclature (SUN) Working Group, 2021). Systemic red flags include persistent fever, characteristic rashes (e.g., syphilitic maculopapular rash), generalized lymphadenopathy, migratory arthralgia, and a clear history of immunosuppression or recent systemic infection (Pihl-Jensen et al., 2021; Schulz et al., 2021). A careful exposure history can further refine risk stratification, including cat contact (suggestive of Bartonella henselae) and tick exposure or travel to endemic areas (suggestive of Borrelia burgdorferi) (Ambika and Lakshmi, 2024). These systemic and exposure clues should be interpreted alongside ocular findings and host immune status rather than in isolation. From an imaging standpoint, brain/orbital MRI showing perioptic enhancement, meningeal involvement, or intracranial inflammatory lesions, together with OCT evidence of inner retinal necrosis or abnormal choroidal thickening, often supports an infectious pathologic process. Importantly, some pathogens can produce prominent signs even in immunocompetent hosts. For example, toxoplasmosis may cause pronounced optic disc swelling and peripapillary infiltration, and brain MRI may reveal periventricular lesions suggestive of concomitant central nervous system involvement (Pihl-Jensen et al., 2021). Visual identification of these subtle infectious red flags is critical to reduce the risk of misclassification and to avoid potentially severe worsening associated with indiscriminate corticosteroid monotherapy (**Figure 3**).

**Table 1** consolidates infection-driven red flags across ocular, systemic, exposure-history, and imaging domains to facilitate early triage and parallel acquisition of “infectious evidence + immune evidence”.

**Table 1:** Infection-driven “red flags” suggestive of direct pathogen invasion in suspected ION.

Domain	Red-flag features (examples)	Typical associated pathogens/conditions (examples)	Ref
Ocular findings	Concomitant uveitis/vitritis; marked vitreous cells or haze	Viral retinitis (e.g., CMV; other Herpesviridae)	(Devilliers et al., 2021; Standardization of Uveitis Nomenclature (SUN) Working Group, 2021)
Ocular findings	Progressive necrotizing retinitis (e.g., “pizza pie” pattern) ± optic disc involvement	CMV; other herpetic necrotizing retinitis	(Devilliers et al., 2021; Standardization of Uveitis Nomenclature (SUN) Working Group, 2021)
Ocular findings	Optic disc swelling with peripapillary infiltrate/neuroretinitis pattern	Toxoplasma; Bartonella henselae	(Pihl-Jensen et al., 2021; Ambika and Lakshmi, 2024)
Ocular findings	Diffuse peripapillary hemorrhages and/or macular edema accompanying inflammatory retinopathy	CMV/herpetic retinitis; severe infectious uveitis/retinitis	(Devilliers et al., 2021; Standardization of Uveitis Nomenclature (SUN) Working Group, 2021)
Systemic/host context	Characteristic rash (e.g., syphilitic maculopapular rash)	Treponema pallidum	(Schulz et al., 2021)
Systemic/host context	Persistent fever; generalized lymphadenopathy (nonspecific—interpret with exposure history and ocular findings)	Systemic infection; opportunistic infection in immunocompromised hosts (context-dependent)	(Jindahra et al., 2020; Ambika and Lakshmi, 2024)
Systemic/host context	Migratory arthralgia (especially with tick exposure/travel history)	Borrelia burgdorferi (context-dependent)	(Ambika and Lakshmi, 2024)
Systemic/host context	Immunocompromised state (e.g., HIV; transplant; long-term immunosuppression) or recent systemic infection	Opportunistic infections (e.g., CMV; fungi; TB)	(Comez, 2019; Jindahra et al., 2020; Ambika and Lakshmi, 2024)
Exposure history	Cat exposure; tick exposure/travel to endemic areas	Bartonella henselae; Borrelia burgdorferi	(Ambika and Lakshmi, 2024)
Neuroimaging (MRI)	Perioptic enhancement; meningeal involvement; intracranial inflammatory lesions; concomitant CNS lesions	Toxoplasma; TB; invasive fungi (context-dependent)	(Comez, 2019; Pihl-Jensen et al., 2021; Ambika and Lakshmi, 2024)
OCT	Supportive OCT changes in infectious neuroretinitis/retinitis (e.g., RNFL edema and inflammatory retinal structural changes)	CMV retinitis; toxoplasmosis (supportive)	(Pihl-Jensen et al., 2021; Standardization of Uveitis Nomenclature (SUN) Working Group, 2021)

Red-flag features are suggestive rather than diagnostic and should be interpreted in the context of host immune status, epidemiologic exposures, and multimodal ocular/neuroimaging findings. The pathogen/condition examples are illustrative and not exhaustive.

ION, infectious optic neuropathy; CMV, cytomegalovirus; MRI, magnetic resonance imaging; OCT, optical coherence tomography; RNFL, retinal nerve fiber layer; TB, tuberculosis; HIV, human immunodeficiency virus; CNS, central nervous system.

### 3.2 Construction of a stratified diagnostic decision tree

**Figure 4:**
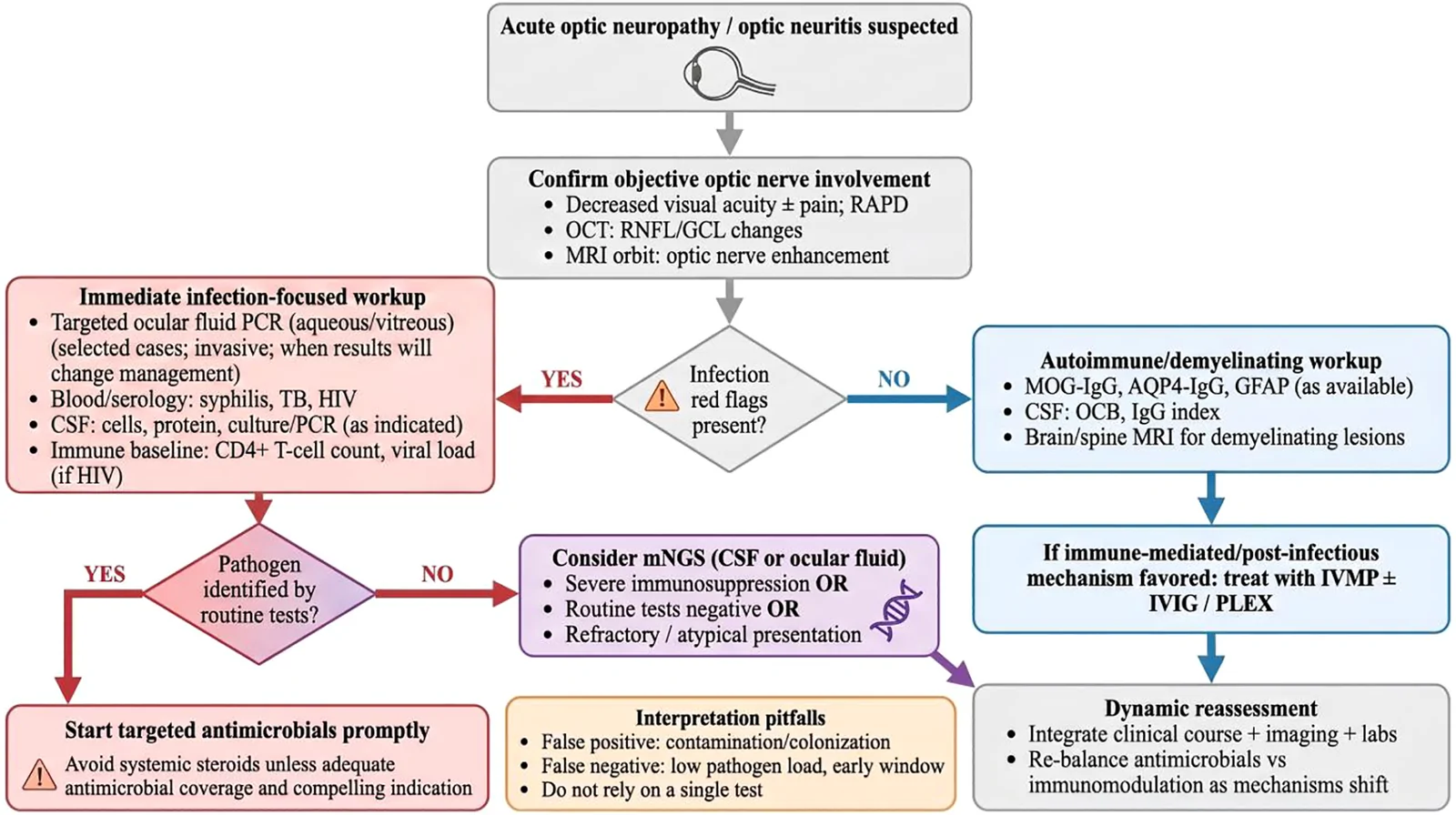
Stratified diagnostic decision tree for suspected infectious optic neuropathy (ION). Step 1 confirms objective optic nerve involvement using clinical and ancillary evidence (e.g., decreased visual acuity ± pain, RAPD; OCT RNFL/GCL changes; orbital MRI optic nerve enhancement). Step 2 triages patients based on infectious red flags into an infection-focused workup versus an autoimmune/demyelinating workup, while highlighting that invasive testing (e.g., aqueous/vitreous PCR) should be considered in selected cases when results are expected to change management. Step 3 emphasizes dynamic reassessment and common interpretation pitfalls (false positives due to contamination/colonization; false negatives due to low pathogen load or early diagnostic windows). mNGS is positioned as an adjunctive test for severe immunosuppression, negative routine testing, or refractory/atypical presentations. (Schematic generated with the assistance of Gemini 3.1 image editor). ION, infectious optic neuropathy; RAPD, relative afferent pupillary defect; OCT, optical coherence tomography; RNFL, retinal nerve fiber layer; GCL, ganglion cell layer; MRI, magnetic resonance imaging; PCR, polymerase chain reaction; TB, tuberculosis; HIV, human immunodeficiency virus; CD4, cluster of differentiation 4; CSF, cerebrospinal fluid; OCB, oligoclonal bands; MOG-IgG, myelin oligodendrocyte glycoprotein IgG; AQP4-IgG, aquaporin-4 IgG; GFAP, glial fibrillary acidic protein; mNGS, metagenomic next-generation sequencing; IVMP, intravenous methylprednisolone; IVIG, intravenous immunoglobulin; PLEX, plasma exchange.

A structured diagnostic decision tree is central to overcoming the differential diagnostic dilemma and typically includes three steps. First, confirm objective evidence of acute optic nerve involvement, including acute visual loss accompanied by a relative afferent pupillary defect (RAPD), abnormal retinal nerve fiber layer thickness on OCT, and optic nerve enhancement on MRI (Pihl-Jensen et al., 2021). Second, triage patients based on red-flag features. If infection is strongly suspected (e.g., fever, concurrent endophthalmitis, immunocompromised state), given the invasive nature of intraocular sampling, intraocular fluid polymerase chain reaction (PCR) testing and other microbiologic evaluations (e.g., blood cultures and pathogen-specific assays) should be considered on a targeted, case-by-case basis, particularly when a specific etiology is suspected and the result is expected to influence clinical management. If the presentation is more suggestive of demyelination, priority should be given to core autoantibody testing, including immunoglobulin G (IgG) antibodies against MOG (MOG-IgG) and AQP4 (AQP4-IgG), as well as antibodies against glial fibrillary acidic protein (GFAP). At this stage, clinicians should avoid the “immune baseline trap” and not rule out infection merely because the patient appears immunocompetent; atypical local manifestations of toxoplasmosis can be misdiagnosed as purely demyelinating (Pihl-Jensen et al., 2021). Third, interpret results carefully and plan reassessment. Cerebrospinal fluid (CSF) findings can provide supportive, non-definitive clues: pleocytosis with elevated protein may favor an infectious or meningoencephalitic process, whereas an isolated OCB pattern may support demyelinating inflammation in the appropriate clinical context (Vlad et al., 2023). Importantly, no single CSF parameter is diagnostic, and results should be integrated with imaging, microbiology, and serology. Clinicians should remain alert to false positives (e.g., specimen contamination or colonizing flora) and false negatives (e.g., pathogen load below the detection threshold) in microbiological testing. Conversely, negative antibody testing should not be used to exclude post-infectious immune-mediated inflammation, particularly during early diagnostic “window” periods. Implementing this decision tree can reduce undue reliance on any single laboratory parameter. Based on red-flag–guided stratification and parallel acquisition of “infectious evidence + immune evidence,” we provide an operational stratified diagnostic workflow (**Figure 4**). This workflow emphasizes selective use of invasive sampling (e.g., aqueous/vitreous PCR) and iterative reassessment as laboratory and imaging results return.

### 3.3 Clinical applications and limitations of mNGS

Within the diagnostic sequence for ION, mNGS is best considered an adjunctive screening tool for complex or severe cases rather than a routine first-line test at initial presentation (ChuanBin et al., 2023). Its primary indications include strong suspicion of infection with failure of empiric antimicrobial therapy, severe immunodeficiency with an unidentified pathogen, and repeatedly negative results on conventional microbiological testing. A major strength of mNGS is its broad, hypothesis-free capability for pathogen detection, particularly for rare viruses or atypical fungi in intraocular or cerebrospinal fluid that may not be covered by targeted PCR panels (Marco and Jessica, 2023). Nevertheless, clinical implementation must be grounded in rigorous contextual assessment to prevent overinterpretation of sequencing outputs. mNGS is susceptible to false positives due to environmental/background microbial signals and to false negatives when specimen volume is limited or pathogen burden is extremely low. Moreover, for pathogens with well-established targeted assays, conventional methods remain the mainstay. For example, despite the heterogeneous phenotypes of toxoplasmosis, diagnosis still relies heavily on pathogen-specific serology or aqueous humor PCR; in this context, mNGS serves only as supportive, confirmatory evidence (Pihl-Jensen et al., 2021). Accordingly, mNGS results should be interpreted through careful triangulation with the clinical phenotype, imaging findings, and conventional laboratory data.

## 4 Treatment strategies

Clinical intervention for ION is far more complex than a one-way choice between “antimicrobial” and “anti-inflammatory” therapy. The crux of decision-making lies in accurately assessing—and continuously recalibrating—the dynamic balance between direct pathogen-mediated injury and host immune-mediated damage. Clinicians should move beyond static, binary thinking (e.g., “steroids are always contraindicated when infection is suspected” or “inflammation warrants indiscriminate immunosuppression”) and instead individualize management based on pathogen characteristics, baseline immune status, and the stage of disease evolution. Among all components of this framework, appropriate use of systemic corticosteroids remains the most controversial, as their effects are highly context-dependent. During active replication of pathogens such as CMV, corticosteroid monotherapy has been reported to be associated with clinical deterioration and may precipitate fulminant necrosis of the retina and optic nerve (Alcibahy et al., 2024). By contrast, observational pediatric data suggest that in children with confirmed post-infectious immune-mediated bilateral optic neuritis, high-dose corticosteroid therapy may improve visual outcomes (Wendel et al., 2020). Even in the setting of IRIS, case-based evidence suggests that the timely introduction of corticosteroids may be important for reversing non-infectious inflammatory compression in selected patients when effective antimicrobial therapy is maintained (Jindahra et al., 2020). For patients presenting with suspected infection but with an unidentified pathogen, antimicrobial therapy remains the cornerstone, and any adjunct corticosteroid use should be guided by compelling indications and effective antimicrobial coverage (Ambika and Lakshmi, 2024). Overall, therapeutic decisions should be grounded in a precise understanding of the evolving pathophysiology rather than rigid disease labels.

### 4.1 Pathogen-directed antimicrobial intervention

Targeted antimicrobial therapy against a confirmed pathogen is the foundation for preventing irreversible axonal injury. The core principles are to select pathogen-sensitive agents and ensure an adequate treatment course. In immunocompetent hosts with CMV-associated optic nerve involvement (often presenting as focal papillitis), case reports suggest that systemic ganciclovir, when appropriately combined with corticosteroids and adequate antiviral coverage, may reduce local inflammation and improve visual function (Melancia et al., 2021). VZV-associated optic neuritis depends critically on early, adequately dosed antiviral therapy; case-based evidence suggests that early initiation of oral valacyclovir in immunocompetent hosts may reverse VZV-induced optic nerve injury and can serve as an alternative to prolonged intravenous therapy in selected cases (Hunt et al., 2021; Gu et al., 2026). For spirochetal and bacterial infections, once syphilitic optic neuropathy is confirmed, treatment should follow neurosyphilis guidance, including high-dose intravenous aqueous penicillin G, with long-term monitoring of cerebrospinal fluid parameters and serologic titers to prevent relapse (Ye et al., 2025). Additional clinically relevant bacterial causes include Bartonella henselae and Borrelia burgdorferi; commonly used treatment principles include doxycycline-based regimens (sometimes combined with rifampin) for Bartonella-associated neuroretinitis/optic nerve involvement, and ceftriaxone- or doxycycline-based therapy for neuroborreliosis-associated optic neuropathy, tailored to systemic and neurologic involvement (Ambika and Lakshmi, 2024).

A major “treatment-related toxicity” dilemma exists in tuberculous optic neuropathy: ethambutol, a first-line anti-tuberculosis agent, is associated with dose- and duration-dependent optic neurotoxicity. Accordingly, patients receiving anti-tuberculosis regimens require close monitoring of visual electrophysiology and visual fields to avoid misattributing drug toxicity to disease progression (Mandal et al., 2021). In contrast, optic nerve abscesses caused by invasive fungi (e.g., Aspergillus and Mucor) are highly aggressive and often require prolonged combination therapy with amphotericin B or voriconazole (months to years), supplemented when indicated by orbital and/or skull-base surgical debridement to reduce local pathogen burden. **Table 2** summarizes commonly used pathogen-directed treatment principles and key clinical caveats relevant to optic nerve involvement, emphasizing context-dependent decision-making rather than guideline-level recommendations.

**Table 2:** Pathogen-directed antimicrobial interventions and key clinical caveats.

Pathogen/infection type	Core pathogen-directed therapy (principles)	Key caveats/special considerations	Ref
Cytomegalovirus (CMV)	Systemic ganciclovir/valganciclovir (as clinically indicated)	Avoid systemic corticosteroid monotherapy during active CMV replication; if corticosteroids are considered, use only under effective antiviral coverage in selected cases	(Melancia et al., 2021; Alcibahy et al., 2024)
Varicella-zoster virus (VZV)	Early, adequately dosed antiviral therapy (e.g., acyclovir/valacyclovir)	Early initiation is critical; oral valacyclovir may be an option for prolonged therapy in selected immunocompetent patients	(Hunt et al., 2021; Gu et al., 2026)
Treponema pallidum (syphilis)	High-dose intravenous aqueous penicillin G (per neurosyphilis approach)	Requires long-term follow-up of serologic titers and, when indicated, CSF parameters to reduce relapse risk	(Ye et al., 2025)
Bartonella henselae (cat-scratch disease)	Commonly used regimens may include doxycycline-based therapy ± rifampin (tailored to systemic/neurologic involvement)	Often presents as neuroretinitis/papillitis; integrate exposure history and serology; avoid misclassification as primary demyelination	(Ambika and Lakshmi, 2024)
Borrelia burgdorferi (Lyme disease/neuroborreliosis)	Ceftriaxone- or doxycycline-based therapy (tailored to neuro involvement and local guidance)	Consider tick exposure/travel history; evaluate for neurologic/systemic involvement (including CSF when indicated)	(Ambika and Lakshmi, 2024)
Mycobacterium tuberculosis	Standard anti-TB therapy	Ethambutol optic neurotoxicity is dose- and duration-dependent; monitor visual function/fields and consider drug toxicity when vision worsens	(Mandal et al., 2021)
Invasive fungi (e.g., Aspergillus, Mucor)	Amphotericin B- and/or azole-based therapy (often prolonged)	Aggressive course possible (e.g., optic nerve/orbital involvement); may require multidisciplinary management and surgical debridement in selected cases	(Ambika and Lakshmi, 2024)

Listed therapies reflect commonly used principles for pathogen-directed management and should be individualized based on disease severity, host immune status, local guidance, and drug accessibility. Where ± is used, it indicates optional adjunct therapy depending on clinical context.

CMV, cytomegalovirus; VZV, varicella-zoster virus; CSF, cerebrospinal fluid; TB, tuberculosis; HIV, human immunodeficiency virus.

### 4.2 Indications for corticosteroid therapy across the pathophysiologic timeline

To minimize the immunosuppressive risks of corticosteroids while maximizing their anti-inflammatory benefits, clinical use should avoid a one-size-fits-all approach and instead adopt a stage-specific, stratified strategy aligned with evolving pathophysiology. In the initial phase—when pathogens are actively replicating—systemic corticosteroids are generally avoided. If considered, they should be introduced only for compelling indications (e.g., severe edema threatening vision), for the shortest possible duration and at the lowest effective dose, and only under stable and effective coverage with potent pathogen-directed antimicrobial therapy. Case reports suggest that in active CMV retinitis with optic nerve involvement, corticosteroid monotherapy can lead to uncontrolled viral replication, and clinical improvement may require prompt discontinuation of corticosteroids and reinitiation of pathogen-specific anti-CMV therapy (Alcibahy et al., 2024). Similarly, in gray-zone cases where the pathogen is unknown but red flags strongly suggest acute infection, indiscriminate high-dose steroid pulse therapy can be harmful; empiric broad-spectrum antimicrobial coverage is typically prioritized first, and corticosteroids should be used with extreme restraint until high-risk, potentially blinding pathogens are reasonably excluded (Ambika and Lakshmi, 2024).

**Figure 5:**
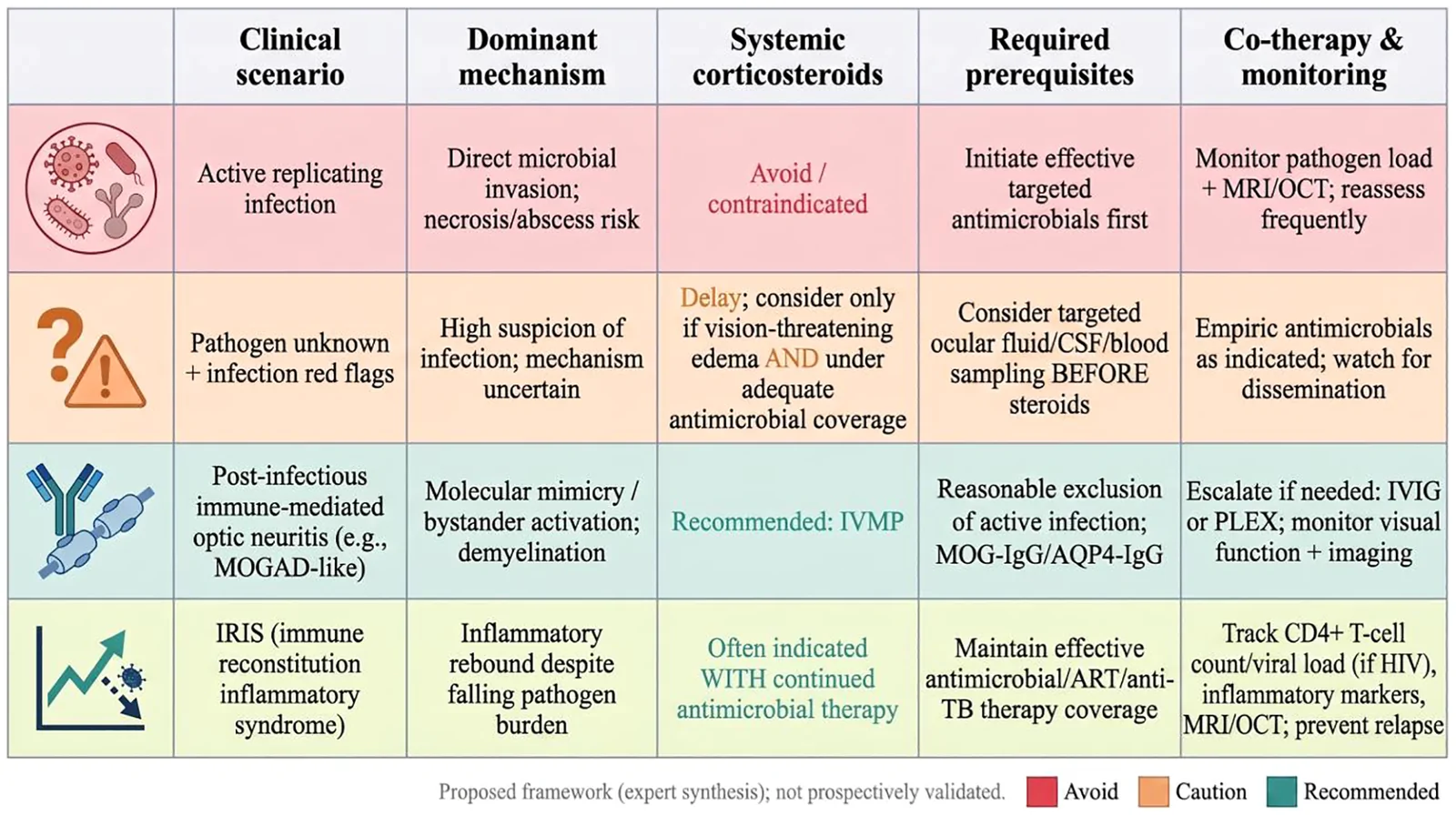
Staged decision matrix for systemic corticosteroid use in infectious optic neuropathy (ION). This matrix summarizes four representative clinical scenarios and aligns systemic corticosteroid decisions with the predominant pathophysiology: (1) active replicative infection, where direct microbial invasion predominates and systemic corticosteroids are avoided/contraindicated as monotherapy while effective targeted antimicrobials and close monitoring (e.g., pathogen load, MRI/OCT) are prioritized; (2) pathogen unknown with infectious red flags, where corticosteroids are delayed and considered only for compelling, vision-threatening inflammatory indications under adequate antimicrobial coverage, ideally after targeted ocular fluid/CSF/blood sampling; (3) post-infectious immune-mediated optic neuritis (e.g., MOGAD-like phenotypes), where corticosteroid pulse therapy (e.g., IVMP) is commonly considered after reasonable exclusion of active infection and integration of immune evidence (e.g., MOG-IgG/AQP4-IgG); and (4) IRIS, where inflammatory rebound occurs despite falling pathogen burden during immune recovery, and corticosteroids may be used in selected cases while maintaining effective antimicrobial/ART/anti-TB therapy coverage and tracking immunologic/inflammatory markers. Color coding (avoid/caution/recommended) reflects pragmatic risk–benefit synthesis rather than guideline-level recommendations. (Schematic generated with the assistance of Gemini 3.1 image editor). ION, infectious optic neuropathy; IRIS, immune reconstitution inflammatory syndrome; MOGAD, myelin oligodendrocyte glycoprotein antibody-associated disease; MOG-IgG, myelin oligodendrocyte glycoprotein IgG; AQP4-IgG, aquaporin-4 IgG; CSF, cerebrospinal fluid; ART, antiretroviral therapy; TB, tuberculosis; HIV, human immunodeficiency virus; IVIG, intravenous immunoglobulin; PLEX, plasma exchange; IVMP, intravenous methylprednisolone (pulse therapy); MRI, magnetic resonance imaging; OCT, optical coherence tomography.

When microbiological testing supports pathogen eradication or durable suppression, and the phenotype aligns with a secondary immune-mediated hit driven by molecular mimicry (i.e., the post-infectious immune-mediated phase), corticosteroids shift from a potential risk factor to a core rescue therapy. In this setting, steroid pulse therapy may be initiated in accordance with standard principles for autoimmune optic neuritis; available clinical evidence, largely from pediatric cohorts, suggests that this approach may improve visual outcomes in post-infectious bilateral optic neuritis (Wendel et al., 2020). In addition, in IRIS, decision-making requires particularly careful calibration: the original antimicrobial regimen should be maintained at an adequate intensity to prevent pathogen rebound, while corticosteroids may be introduced to mitigate the paradoxical inflammatory surge driven by abrupt immune recovery. Case reports and small series suggest that corticosteroids can relieve perioptic inflammatory compressive symptoms in HIV-associated CMV-associated IRIS and in tuberculosis-associated paradoxical inflammatory worsening, including tuberculosis-associated IRIS in immunocompromised hosts (Quinn et al., 2020). Because the benefit–risk profile of corticosteroids depends heavily on disease stage and antimicrobial coverage, and because decision-making is often supported by heterogeneous evidence, we summarize common clinical scenarios in a practical decision matrix (**Figure 5**). This matrix represents a proposed, mechanism-informed framework synthesized from disparate evidence and has not yet been prospectively validated. The matrix is intended to support bedside risk–benefit reasoning when antimicrobial and immunomodulatory priorities compete, and it should be applied with stage-aware monitoring.

### 4.3 Second-line immunomodulation and neuroprotection for refractory disease

When patients are refractory to first-line pathogen-directed therapy plus conventional corticosteroid regimens, or when corticosteroids are contraindicated, early consideration of second-line immunomodulatory and neuroprotective strategies may be warranted. For refractory post-infectious immune-mediated optic neuritis, IVIG and plasma exchange (PLEX) are commonly used rescue therapies to help remove circulating pathogenic factors and interrupt inflammatory cascades. This is particularly relevant in severe MOGAD or AQP4-positive optic neuritis, in which PLEX may help reduce the levels of circulating pathogenic autoantibodies. Comparative studies suggest that during acute attacks, patients with MOGAD may demonstrate higher response rates to corticosteroids and PLEX than patients with AQP4-IgG–positive NMOSD (Rempe et al., 2021).

In recent years, targeted biologics have also been used in selected refractory settings. For example, anti–interleukin-6 receptor (IL-6R) monoclonal antibodies (e.g., tocilizumab) have been reported to attenuate inflammatory crises in refractory IRIS and NMOSD in some studies (Song et al., 2020). Although B-cell depletion therapy (e.g., rituximab) is effective in other autoimmune ocular diseases (such as moderate-to-severe active Graves’ ophthalmopathy) (Vannucchi et al., 2021), extending its routine use to post-infectious optic neuritis warrants caution, with vigilance for reactivation of latent infections such as John Cunningham virus (JCV) and hepatitis B virus (HBV) due to cumulative immunosuppression.

Finally, beyond controlling inflammation and infection, neuroprotective interventions targeting axons themselves remain clinically relevant. In a preclinical rat model of ethambutol-induced optic neuropathy, citicoline has been reported to mitigate retinal ganglion cell loss and retinal nerve fiber layer damage, suggesting potential neuroprotective effects relevant to patients receiving antituberculosis therapy (Nusanti et al., 2022).

Overall, rescue strategies for refractory cases should seek an optimal balance across three domains: pathogen suppression, immune homeostasis remodeling, and neuronal repair.

## 5 Future research directions

The clinical management of ION—particularly severe entities such as CMV retinitis with optic nerve involvement—has long been constrained by limited high-quality evidence. Most therapeutic decisions remain based on retrospective case series or expert opinion. For example, in non-HIV immunocompromised patients (e.g., solid-organ transplant recipients and patients with malignancies), CMV infection shows marked heterogeneity in both phenotype and treatment response, yet large prospective cohorts for systematic characterization remain lacking (Sadik et al., 2021; Passarin et al., 2024). This evidence gap has fueled multiple clinical controversies, including when to initiate pathogen-directed testing in immunocompetent hosts and how to balance antiviral therapy with immunomodulation in immune recovery uveitis (IRU) (Rodrigues et al., 2024). To address these unmet needs, future research should be advanced across four domains: diagnostic technologies, biomarkers, clinical trials, and fundamental mechanisms.

### 5.1 Technical evaluation and standardization in diagnostics

Evaluating mNGS in clinical screening pathways. mNGS may have potential utility for atypical ION and for cases with negative conventional PCR testing. However, existing standards still rely heavily on dual validation of clinical features and aqueous/vitreous PCR (Standardization of Uveitis Nomenclature (SUN) Working Group, 2021). Given that other pathogens, such as T. pallidum, can also produce CMV-like necrotizing retinal changes (Rissotto et al., 2024), the broad, hypothesis-free detection capability of mNGS may be particularly valuable. Nevertheless, its high cost and susceptibility to false positives (colonization/contamination)—especially in HIV-negative patients, where presentations are often highly atypical (Sadik et al., 2021; Wang I. et al., 2024)—underscore the need for multicenter prospective cohort studies. Such studies should systematically evaluate the cost-effectiveness, sensitivity, and specificity of mNGS across diverse host populations to clarify its optimal positioning as a first-line screening tool versus a second-line adjunct.

Standardizing and quantifying multimodal imaging parameters. Inter-center consistency in imaging-based diagnosis remains suboptimal. For instance, retinal pigment epithelium atrophy or outer retinal disruption on OCT—observed in congenital CMV infection or antiviral drug-related toxicity—has often been described qualitatively rather than quantitatively (Kapoor et al., 2024; Kozek et al., 2024). Future work should integrate OCT, optical coherence tomography angiography (OCTA), and brain/orbital MRI to establish standardized quantitative imaging acquisition protocols (e.g., defining thresholds for retinal nerve fiber layer (RNFL) thickness, deep capillary plexus perfusion density, and specific patterns of optic nerve enhancement on MRI). These efforts would facilitate the development of objective imaging biomarkers for more precise stratification and prognostic prediction.

### 5.2 Development and validation of multidimensional biomarkers

Defining OCTA microvascular parameters as mechanism-informative biomarkers. OCTA may become a noninvasive tool for distinguishing underlying mechanisms. Preliminary observations suggest that direct infection, such as congenital CMV, more readily causes substantial loss of the choriocapillaris (Kozek et al., 2024), whereas ischemic injury, such as non-arteritic anterior ischemic optic neuropathy (NAION), more commonly manifests as a steep decline in superficial microvascular density (Dhiman et al., 2020; Phuljhele et al., 2023). By contrast, post-infectious MOGAD or IRU-like immune-mediated injuries may exhibit distinct patterns of superficial/deep vascular dilation or leakage. Large-scale studies are needed to define pathogen- and stage-specific OCTA “microvascular fingerprints.”

Clarifying the prognostic value of neurofilament light chain (NfL). Serum or cerebrospinal fluid NfL, a sensitive marker of axonal injury, has been associated with final visual outcomes in demyelinating optic neuritis and acute NAION (Shah et al., 2025). Future studies should extend NfL to ION to determine whether acute-phase serum NfL (sNfL) peaks can quantitatively reflect irreversible optic nerve damage caused by pathogens such as CMV or T. pallidum, and whether NfL can serve as a predictor of response to antiviral and immunomodulatory therapy.

Profiling intraocular cytokines to differentiate infection from immune-mediated injury. Molecular signatures of the intraocular microenvironment may help distinguish infection from autoimmunity. CMV retinitis and IRIS have been reported to be accompanied by upregulation of interleukin-6 (IL-6) and interleukin-8 (IL-8) in aqueous humor (Tang et al., 2021), whereas autoimmune optic neuritis such as MOGAD may show a T helper 17 (Th17)–skewed profile [elevated IL-6/interleukin-17 (IL-17)]. Future studies should leverage standardized platforms (e.g., multiplex bead immunoassays) to define clinically actionable thresholds for ratios, such as interleukin-10 (IL-10)/IL-6, to differentiate active infection from secondary immune phases, thereby supporting the precision use of biologics such as tocilizumab, which targets IL-6R (Nguyen et al., 2024).

### 5.3 Building an evidence base for therapeutics

Establishing high-quality multicenter real-world registries. Given the rarity of the disease, multicenter registry studies are essential to accumulate robust evidence. Current treatment options for CMV retinitis include systemic and local antiviral agents, adoptive immunotherapy with CMV-specific cytotoxic T lymphocytes (CMV-CTLs), and CMV immune globulin (CMVIG). However, real-world effectiveness varies substantially among complex hosts, such as stem cell transplant recipients (Gupta et al., 2021; Mo et al., 2022; Chiu et al., 2023). Registries can provide harmonized evaluations of long-term effectiveness and delayed complications (e.g., retinal detachment) (Puri and Kumar, 2024; Rathinam et al., 2026), and can also examine relapse control rates associated with early corticosteroid use in IRU (Rodrigues et al., 2024), thereby providing a data foundation for iterative guideline updates.

Conducting randomized controlled trials (RCTs) targeting the post-infectious immune-mediated phase. Precisely calibrating the degree of immunosuppression during IRIS or post-infectious MOGAD remains a major unmet need. Current advances—such as case-level reports of tocilizumab used to treat IRU-associated macular edema in patients with contraindications to corticosteroids—remain preliminary and should be interpreted with caution (Nguyen et al., 2024). Future multinational, multicenter RCTs are needed to compare high-dose steroid pulse therapy, IVIG, and combination regimens within well-defined pathophysiologic strata (e.g., confirmed post-infectious MOGAD), using outcomes such as visual salvage rates and inflammatory resolution. Such trials are critical for building higher-level evidence to balance immunosuppression against the risk of infection reactivation.

### 5.4 Deeper decoding of fundamental mechanisms

Leveraging single-cell multi-omics to resolve the local immune microenvironment. Conventional histology has limited ability to capture the spatiotemporal dynamics of lesional microenvironments (Song et al., 2025). Single-cell RNA sequencing (scRNA-seq) can enable dissection of macrophage/microglial polarization states (M1/M2) within CMV or demyelinating lesions at single-cell resolution and facilitate tracking of clonal expansion trajectories of autoreactive T and B cells and local antibody repertoires. These approaches may pinpoint key signaling pathways that drive IRIS or molecular mimicry and identify actionable targets for next-generation immunotherapies.

Developing high-fidelity animal disease models. Overcoming translational bottlenecks requires improved *in vivo* platforms. Existing pathogen-only infection models (e.g., murine CMV models) do not recapitulate the complex post-infectious autoimmune cascades observed in humans. Future efforts should prioritize transgenic models that simulate the full course of molecular mimicry (e.g., by inducing disease in humanized MOG mice with specific viral peptides) or immune reconstitution models that reproduce IRIS-like features. High-fidelity models will not only validate targets identified via scRNA-seq but will also serve as critical testbeds for evaluating the safety and efficacy of emerging IL-6R antagonists or complement inhibitors.

## 6 Conclusion

Clinical management of ION is increasingly shifting from experience-driven practice toward a mechanism-guided approach. The disease is not a static binary opposition between “infection” and “autoimmunity,” but rather a dynamic trajectory in which direct pathogen invasion and secondary immune-mediated injury may predominate at different time points and can overlap throughout the disease course. This recognition argues for moving beyond a delayed, passive diagnosis-of-exclusion strategy and instead implementing an upfront, precision stratification system anchored in red-flag features, mNGS, and pathogen-specific and autoimmune antibody profiles. At the most contentious decision point—systemic corticosteroid use—treatment should be aligned with the underlying pathophysiologic stage (active infection, post-infectious immune response, or immune reconstitution), with dynamic matching of timing and dose to the host immune microenvironment to mitigate the dual risks of pathogen dissemination and irreversible inflammatory axonal injury. Given the current paucity of high-level evidence in this field, future research should prioritize multicenter prospective cohorts and RCTs to validate emerging biomarkers such as OCTA microvascular metrics and serum NfL, and to define standardized immunomodulatory windows across different pathogen contexts. Ultimately, efficient interdisciplinary collaboration and continuous reassessment of the pathogen–host immune balance may offer the greatest opportunity to preserve and restore vision in affected patients.
